# Supplementary material for: Silicone-Thioxanthone: A Multifunctionalized Visible Light Photoinitiator with an Ability to Modify the Cured Polymers
Source: Polymers (Basel). 2019 Apr 16;11(4):695. doi: 10.3390/polym11040695 (PMC6523732; doi:10.3390/polym11040695)
Supplement: Supplementary file 1 [file polymers-11-00695-s001.pdf]

Supporting Information

Silicone-thioxanthone: A multifunctionalized visible light photoinitiator with an ability to modify the cured polymers

Qingqing Wu #, Wen Liao #, Ying Xiong, Jianjing Yang, Zhen Li, Hongding Tang *

Engineering Research Center of Organosilicon Compounds & Materials, Ministry of Education, College of Chemistry and Molecular Sciences, Wuhan University, Wuhan, 430072, P. R. China

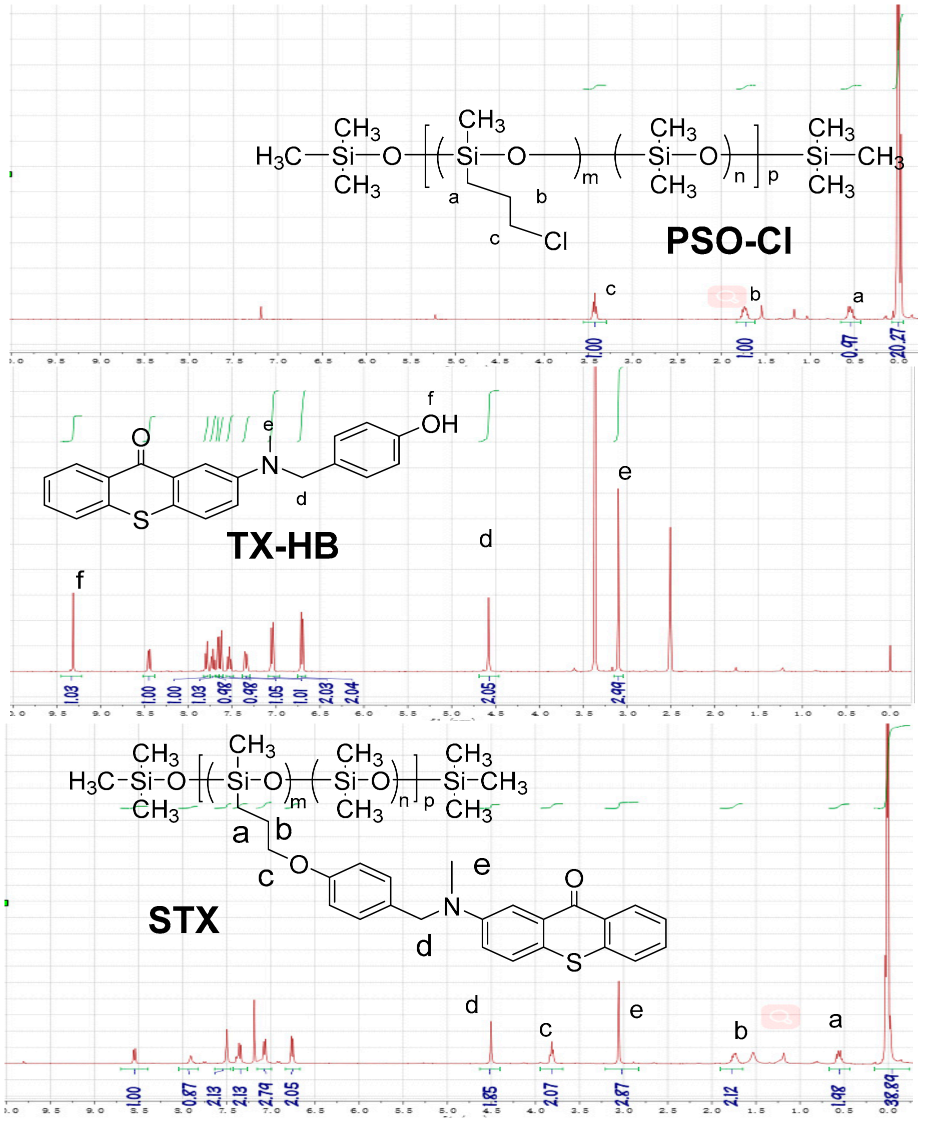

Figure S1. ^{1}H NMR spectra of PSO-Cl ($CDCl_3$), TX-HB (DMSO) and STX ($CDCl_3$).

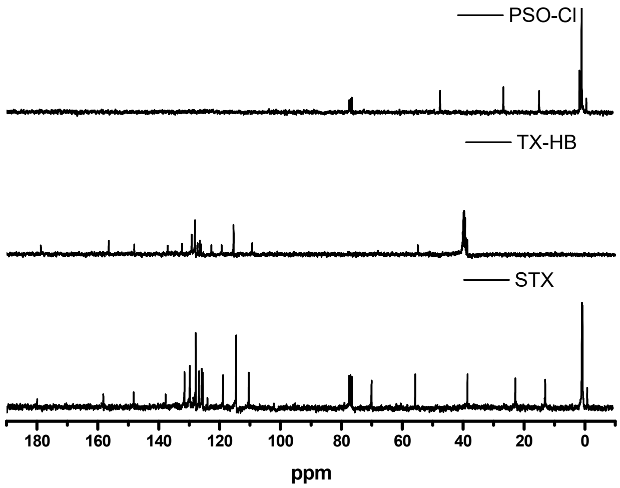

Figure S2. ^{13}C NMR spectra of PSO-Cl ($CDCl_3$), TX-HB (DMSO) and STX ($CDCl_3$).

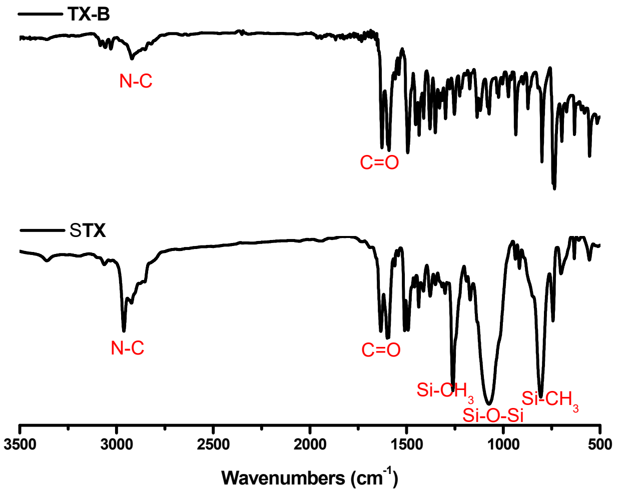

Figure S3. FTIR spectra of TX-B and STX.

STX (0.0074 g) was dissolved in THF and diluted to 50.00 mL. Then the UV-vis spectrum of STX was measured to determine the content of ATX moiety (Fig. S5). It is calculated according to Lambert-Beer law:

$$A = \varepsilon bC = \frac{\varepsilon bn}{V} \qquad (1)$$

where molar extinction coefficient of TX-B ($\varepsilon = 4848$ $L \cdot mol^{-1} \cdot cm^{-1}$) is used as a standard of thioxanthone group, b is optical path length, which is 1 cm, and V is 50

mL. The number mole of thioxanthone group in STX is 8.37×10^{-6}. The average number of ATX moiety in STX is calculated to be about 3.3.

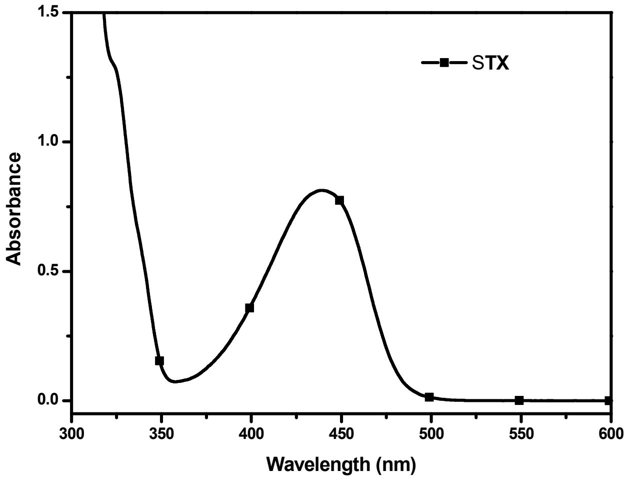

Figure S4. UV-vis of STX in THF.

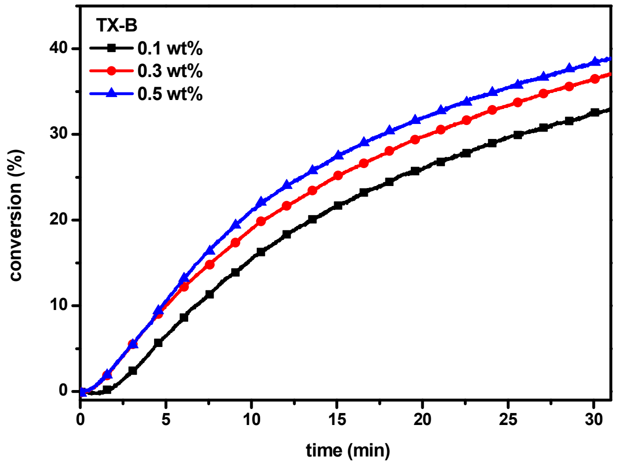

Figure S5. Conversion-time and rate-time for polymerization of TMPTA in THF (THF % = 25 wt%) in laminate initiated by TX-B in different concentrations.

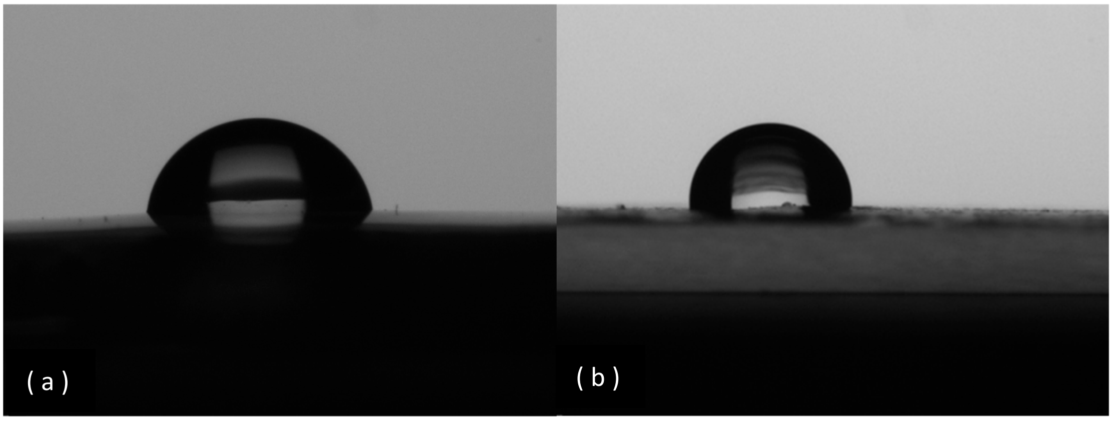

Figure S6. The contact angle images of the polymer film initiated by photoinitiator; (a): TX-B (81.8°); (b): STX, [STX] = 6 × 10^{-5} mol/g, (102.3°).

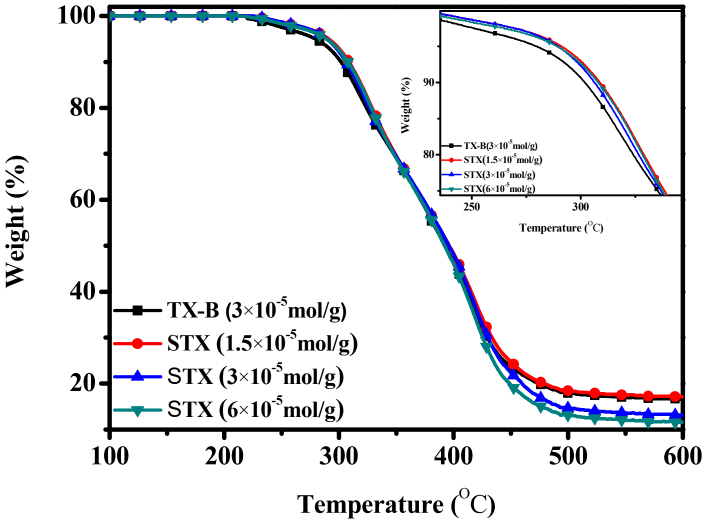

Figure S7. TGA curves of polymer films initiated by TX-B and STX.

Table S1. The thermal data of TX-B and STX.

PI	Temperature		Char (%)
	T_5 (°C)	T_{50} (°C)	
**TX-B**	273	326	0.01
**STX**	229	373	1.29

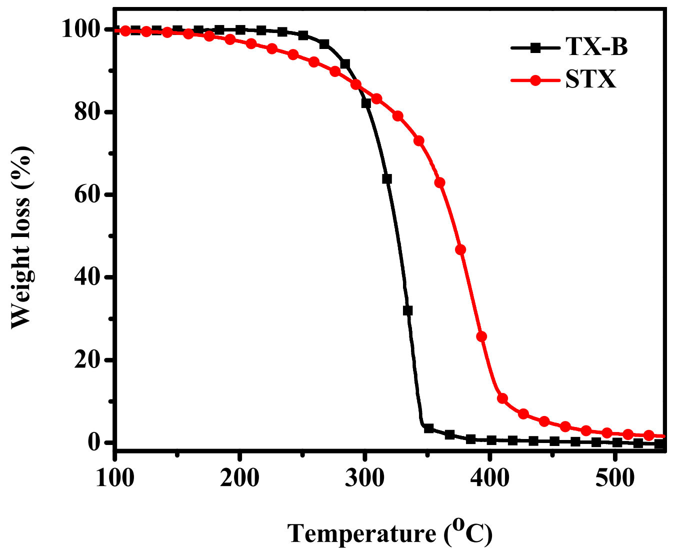

Figure S8. TGA curves of TX-B and STX.

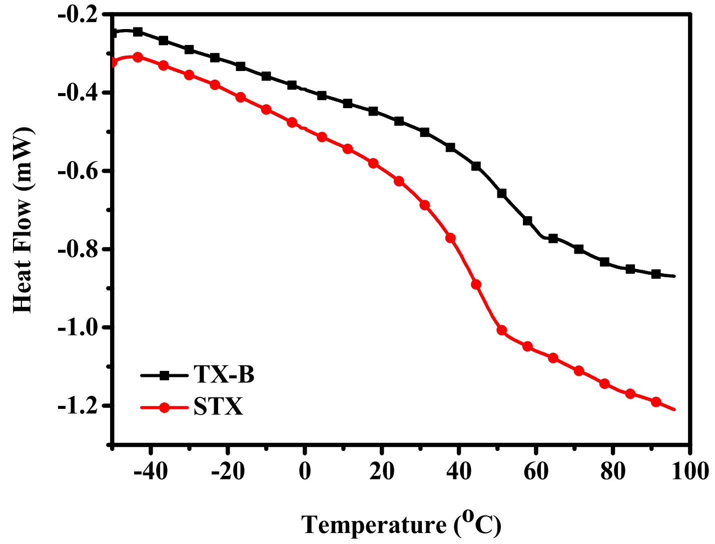

Figure S9. DSC curves of cured PUA films initiated by TX-B and STX ([TX-B] = 3 × 10^{-5} mol g^{-1}, [STX] = 3 × 10^{-5} mol g^{-1}). [STX] refers to the molar concentration of the ATX moiety.
